# Anti-Interleukin-22-Neutralizing Antibody Attenuates Angiotensin II-Induced Cardiac Hypertrophy in Mice

**DOI:** 10.1155/2017/5635929

**Published:** 2017-11-22

**Authors:** Jing Ye, Ling Liu, Qingwei Ji, Ying Huang, Ying Shi, Lei Shi, Jianfang Liu, Menglong Wang, Yao Xu, Huimin Jiang, Zhen Wang, Yingzhong Lin, Jun Wan

**Affiliations:** ^1^Department of Cardiology, The People's Hospital of Guangxi Zhuang Autonomous Region, Nanning, China; ^2^Department of Cardiology, Hubei Key Laboratory of Cardiology, Renmin Hospital of Wuhan University, Cardiovascular Research Institute, Wuhan University, Wuhan 430060, China; ^3^Emergency & Critical Care Center, Beijing Institute of Heart, Lung, and Blood Vessel Diseases and Beijing Anzhen Hospital, Capital Medical University, Beijing 100029, China

## Abstract

**Background:**

Interleukin- (IL-) 22 is considered a proinflammatory cytokine. Recent evidence has demonstrated that it plays a role in cardiovascular diseases. In the recent study, we investigate whether IL-22 is involved in cardiac hypertrophy.

**Methods:**

Angiotensin II was used to build hypertrophy model and the IL-22 and IL-22 receptor 1 (IL-22R1) levels in heart tissue were measured. In addition, angiotensin II-treated mice received an injection of anti-IL-22-neutralizing antibody (nAb) to investigate the effects of IL-22 nAb on myocardial hypertrophy, cardiac function, and cardiac fibrosis; the activation of the signaling pathway and the prohypertrophic inflammatory cytokine mRNA levels was detected. Furthermore, the effect of IL-22 nAb on angiotensin II-induced hypertrophy in vitro was also determined.

**Results:**

IL-22 and IL-22R1 levels were significantly increased after angiotensin II infusion. Anti-IL-22 nAb significantly alleviated the severity of hypertrophy, prevented systolic and diastolic abnormalities, reduced cardiac fibrosis, STAT3 and ERK phosphorylation, and downregulated the mRNA expression of IL-17, IL-6, IL-1*β*, IFN-*γ*, and TNF-*α*. In addition, IL-22 nAb attenuated angiotensin II-induced hypertrophy in H9C2 cells.

**Conclusion:**

Our data demonstrated that neutralization of IL-22 alleviated angiotensin II-induced cardiac hypertrophy. The downregulation of IL-22 may be a novel therapeutic strategy to prevent cardiac hypertrophy.

## 1. Introduction

Pathological cardiac hypertrophy, which is characterized by myocardial enlargement and abnormal fibrosis of the extracellular matrix, is a compensatory stage of chronic heart failure (CHF). Pathological cardiac hypertrophy can be stimulated by various pathological factors [1, 2], and when it progresses to CHF, it may cause serious clinical complications, such as malignant arrhythmia and sudden cardiac death. Although clinical drug therapy is beneficial for improving the quality of life and survival rate of CHF patients, the prognosis is still poor [3]. Therefore, preventing and postponing the development of cardiac hypertrophy are important therapeutic strategies for CHF [4].

IL-22 belongs to the IL-10 cytokine family and is primarily secreted by lymphocytes [5, 6]. IL-22 plays a proinflammatory role and participates in a variety of diseases by increasing levels of inflammatory mediators [7, 8]. However, accumulating evidence has demonstrated that IL-22 also has an anti-inflammatory effect and ameliorates diseases by reducing inflammation [9, 10]. This phenomenon was also observed in cardiovascular diseases. The upregulation of IL-17, IL-6, and TNF-*α* and the downregulation of IFN-*γ* were observed after treatment with mouse anti-IL-22 nAb in a coxsackievirus B3- (CVB3-) induced acute viral myocarditis (AVMC) mouse model [11], and this effect was prevented in IL-17A-deficient mice [12]. Genetic deletion of IL-22 decreased IL-6 secretion in high-fat-fed apolipoprotein E knockout mice [13].

Although clinical data have demonstrated that compared to the control group, higher serum IL-22 levels were observed in CHF patients with an NYHA class of II and III [14], the involvement of IL-22 in cardiac hypertrophy is still unknown, and the pathogenesis remains to be clarified. Therefore, this study aimed to investigate the role of IL-22 in cardiac hypertrophy mice.

## 2. Materials and Methods

### 2.1. Animals and Animal Models

Male C57BL/6 mice (HFK Bioscience, Beijing, China) aged 9-10 weeks with a weight of 25.5–27.5 g were housed in the pathogen-free mouse room of Renmin Hospital of Wuhan University. Chronic angiotensin II infusion was used to establish the mouse hypertrophy model. IL-22 and IL-22R1 expressions in heart tissue were detected on weeks 1, 2, and 4, and saline infusion mice were used to determine the baseline level (*n* = 4 for each group). In addition, angiotensin II-induced cardiac hypertrophy mice received daily intraperitoneal injections of 50 *μ*l PBS, 1.25 *μ*g mouse anti-IL-22 nAb [9], or an equivalent amount of isotype IgG, while saline infusion mice received PBS as a control (*n* = 8 for each group). At 4 weeks after angiotensin II infusion, all mice were euthanized, and the hearts were dissected and weighed to calculate the heart weight/body weight (HW/BW) and heart weight/tibia length (HW/TL) ratios. All the experimental procedures were performed in accordance with the institutional guidelines of the animal care and use committee of Renmin Hospital of Wuhan University, and this study was approved by the ethics committee of the People's Hospital of Guangxi Zhuang Autonomous Region (Nanning, China) and Renmin Hospital of Wuhan University (Wuhan, Hubei, China).

### 2.2. Osmotic Mini Pump Implantation

After the mice were anesthetized with 2% isoflurane, angiotensin II (1.4 mg/kg/day, Enzo Life Sciences Inc., Farmingdale, USA) [15] or saline was infused by osmotic mini pumps (Alzet model 2001/2002/2004, California, USA) as previously described [16].

### 2.3. Echocardiography

Four weeks after implantation with osmotic mini pumps, mice were anesthetized with 2% isoflurane, and the structure and function of the left ventricle (LV), including the heart rate (HR), LV end-systolic diameter (LVESD), LV end-diastolic diameter (LVEDD), LV posterior wall thickness (LVPWD), end-diastolic ventricular septal thickness (IVSD), ejection fraction (EF), and fractional shortening (FS), were measured by the MyLab™ 30CV ultrasound system (Esaote SpA, Genoa, Italy) equipped with a 10 MHz linear array ultrasound transducer.

### 2.4. Histological Analysis

Hearts were isolated and immediately arrested in diastole with 10% KCl. After being fixed with 4% neutral paraformaldehyde for 7 days, the hearts were embedded in paraffin, cut into 4-5 mm slices and mounted onto slides. Hematoxylin and eosin (H&E) staining was used for histopathological analysis, and at least 100 cells of each group were used to assess the cross-sectional area (CSA) of myocardial cells. Picrosirius red (PSR) staining was performed to detect the collagen expression level. Mouse anti-IL-22 antibody was used to mark the IL-22 expression in the heart.

### 2.5. Western Blot Analysis

The LV tissue was lysed in radioimmunoprecipitation assay (RIPA) lysis buffer, and the total protein was extracted and detected with a BCA Protein Assay Kit (Thermo Fisher Scientific, MA, USA). Approximately 30 *μ*g of total protein was separated by electrophoresis on Laemmli sodium dodecyl sulfate (SDS) polyacrylamide gels. After electrophoresis, the samples were transferred to Immobilon-FL PVDF membranes (Millipore, USA). The membranes were blocked with 5% nonfat milk and then incubated with anti-IL-22, anti-IL-22R1 (both from Abcam, Cambridge, England), anti-atrial natriuretic peptide (ANP), anti-B-type natriuretic peptide (BNP), anti-*β*-myosin heavy chain (*β*-MHC, triple from Santa Cruz, Dallas, TX, USA), anti-STAT3, anti-phospho-STAT3, anti-ERK, anti-phospho-ERK, anti-c-Jun N-terminal kinase (JNK), anti-phospho-JNK, anti-P38, anti-phospho-P38, and anti- glyceraldehyde-3-phosphate dehydrogenase (GAPDH, nine above from Cell Signaling Technology, Boston, USA) antibodies at 4°C overnight. The secondary antibodies were incubated at room temperature for 1 hour. The blots were scanned using a two-color infrared imaging system (Odyssey; LI-COR Biosciences, Lincoln, NE, USA).

### 2.6. Quantitative Polymerase Chain Reaction (RT-qPCR)

The total mRNA of LV tissue was extracted with TRIZOL Reagent, and a reverse transcription kit was used to synthesize cDNA according to the manufacturer's instructions. PCR amplification was performed using a LightCycler 480 SYBR Green Master Mix (Roche, Mannheim, Germany). The relative mRNA expression levels of fibronectin, connective tissue growth factor (CTGF), collagen I*α*, collagen III*α*, transforming growth factor- (TGF-) *β*, IL-1*β*, IL-6, IL-17, IFN-*γ*, TNF-*α*, and IL-22R1 were investigated, and the mRNA expression was normalized to that of GAPDH. The RT-qPCR primer sequences are shown in Table 1.

### 2.7. Measurement of Serum Creatinine and Blood Urea Nitrogen

Blood samples were collected and centrifuged at 200*g* for 10 min, and the serum with blood samples was isolated. The serum blood urea nitrogen (BUN) and creatinine levels were measured using a kit from Bioassay Systems (Hayward, CA) following the manufacturer's protocol.

### 2.8. Cell Culture

The H9C2 cells are resuscitated and grown in high-glucose DMEM supplemented with 10% FBS, 100 U/ml penicillin, and 100 mg/ml streptomycin in a humidified incubator (Sanyo Electric Co., Japan) in 5% CO_2_ at 37°C. After cultured with serum-free DMEM for 12 h, the H9C2 cells were treated with saline + PBS, saline + IL-22 nAb (5 *μ*g/ml) [17], Ang II (1 *μ*M) + PBS, Ang II + IL-22 nAb, respectively. The cells were incubated for the indicated time.

### 2.9. Statistical Analyses

All data are expressed as the mean ± standard deviation (SD). Differences between the means of two groups were compared by Student's *t*-test. Differences between the means of multiple groups were compared by one-way analysis of variance (ANOVA) followed by Tukey's multiple comparison test. A value of *p* < 0.05 was considered significant.

## 3. Results

### 3.1. IL-22 and IL-22R1 Levels Are Increased in Angiotensin II-Induced Hypertrophic Hearts

**Figure 1:**
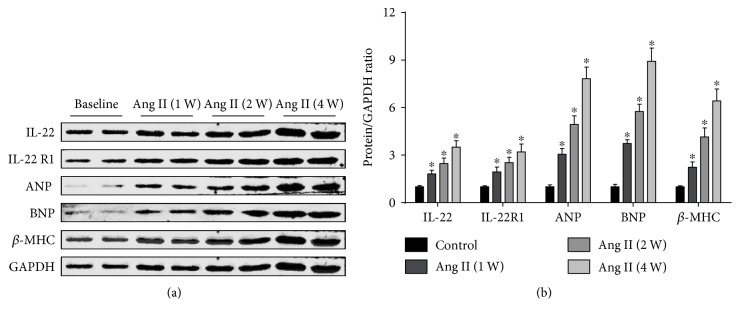
Effect of chronic angiotensin II infusion on IL-22/IL-22R1 protein levels. (a) Representative and (b) quantitative expression of IL-22, IL-22R1, ANP, BNP, and *β*-MHC in LV tissue of baseline and angiotensin II-infused mice. *n* = 4 for each group. ^∗^*p* < 0.05 versus baseline level.

Compared to baseline levels, the IL-22 and IL-22R1 protein levels were significantly increased in angiotensin II-induced hypertrophic LV tissue (Figures 1(a) and 1(b)). The increase occurred almost in parallel with the upregulation of hypertrophy marker proteins, including ANP, BNP, and *β*-MHC (Figures 1(a) and 1(b)).

### 3.2. IL-22 nAb Alleviates Angiotensin II-Induced Cardiac Hypertrophy

**Figure 2:**
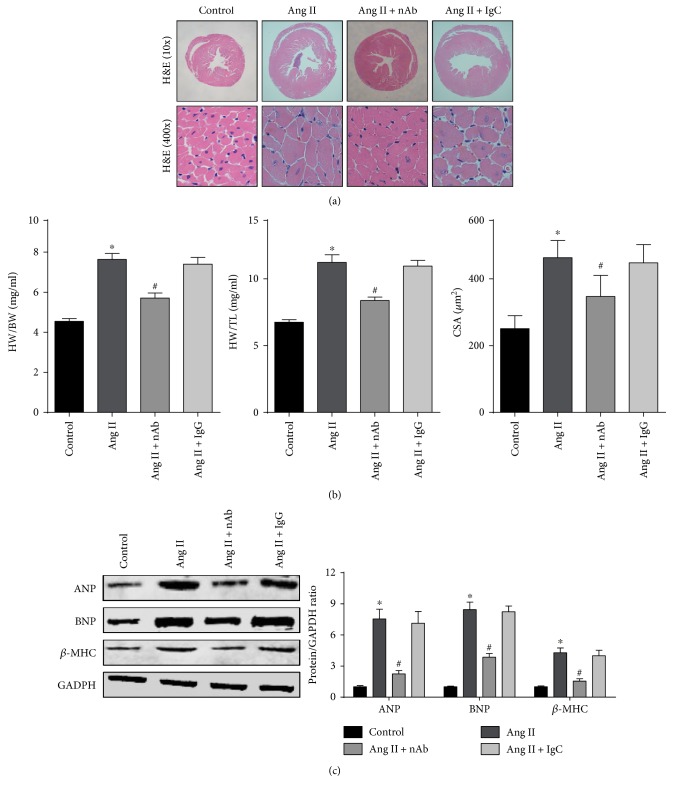
Effect of anti-IL-22 nAb on angiotensin II-induced cardiac hypertrophy. (a) Gross hearts stained with H&E in each group, *n* = 4-5 for each group. (b) Values of the HW/BW, HW/TL ratios and myocyte CSA in control and chronic angiotensin II infusion groups, *n* = 8 for each group. (c) Representative and quantitative expression of ANP, BNP, and *β*-MHC in the LV of the four groups. *n* = 4 for each group. ^∗^*p* < 0.05 versus control. ^#^*p* < 0.05 versus Ang II group.

Histological analysis and Western blots were used to evaluate the effect of anti-IL-22 nAb on cardiac remodeling. H&E staining of heart tissue confirmed that cardiac hypertrophy induced by angiotensin II was prevented by mouse anti-IL-22 nAb (Figure 2(a)). Treatment with mouse anti-IL-22 nAb resulted in a reduction in the HW/BW and LW/TL ratios and the CSA in angiotensin II-induced cardiac hypertrophy mice (Figure 2(b)). Furthermore, the angiotensin II-induced increase of hypertrophy marker proteins, such as ANP, BNP, and *β*-MHC, was mitigated by mouse anti-IL-22 nAb (Figure 2(c)). Isotype IgG had no effect on angiotensin II-induced cardiac hypertrophy (Figure 2(a)–2(c)).

### 3.3. IL-22 nAb Improves Impaired Cardiac Function in Mice

The structure and function of the LV were measured by echocardiography to determine the effect of anti-IL-22 nAb on the progression of angiotensin II-induced cardiac dysfunction. The results showed that angiotensin II infusion for 4 weeks induced increases in the LVESD, LVEDD, LVPWD, and IVSD and reductions of the EF and FS. Angiotensin II-mediated exception of structure and function of the LV was alleviated by mouse anti-IL-22 nAb treatment. No significant difference in HR was observed among the four groups. Compared to the angiotensin II group, no significant change in cardiac dysfunction was observed in the angiotensin II + IgG group. The echocardiography data of each group are shown in Table 2.

### 3.4. IL-22 nAb Protects against Angiotensin II-Induced Fibrotic Response in the Heart

**Figure 3:**
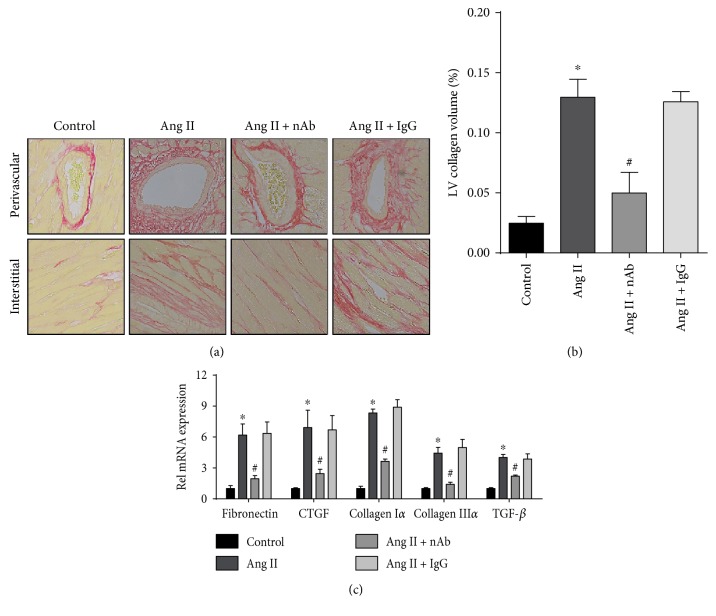
Effect of the anti-IL-22 nAb on angiotensin II-induced cardiac fibrosis. (a) Cardiac interstitial fibrosis was determined in the heart sections from each group by PSR straining, *n* = 4-5 for each group. (b) Ratios of fibrotic areas on histological sections were quantified by an image-analysis system. (c) The mRNA expression levels of fibronectin, CTGF, collagen I*α*, collagen III*α*, and TGF-*β* in the myocardium of each group were determined by RT-qPCR, *n* = 3-4 for each group. ^∗^*p* < 0.05 versus control. ^#^*p* < 0.05 versus Ang II group.

Interstitial fibrosis and the fibrotic response of perivascular and cardiac interstitial collagen deposition were detected by PSR staining to evaluate the effect of anti-IL-22 nAb on cardiac fibrosis. The results showed that anti-IL-22 nAb mitigated the angiotensin II-induced cardiac fibrosis area ratio, and isotype IgG did not affect angiotensin II-induced cardiac fibrosis (Figures 3(a) and 3(b)). Similar results were obtained for the mRNA expression levels of mediators of fibrosis, including fibronectin, CTGF, collagen I*α*, collagen III*α*, and TGF-*β* (Figure 3(c)).

### 3.5. IL-22 nAb Inhibits Activation of the ERK and STAT3 Pathways and the mRNA Expression of Prohypertrophic Inflammatory Cytokines

**Figure 4:**
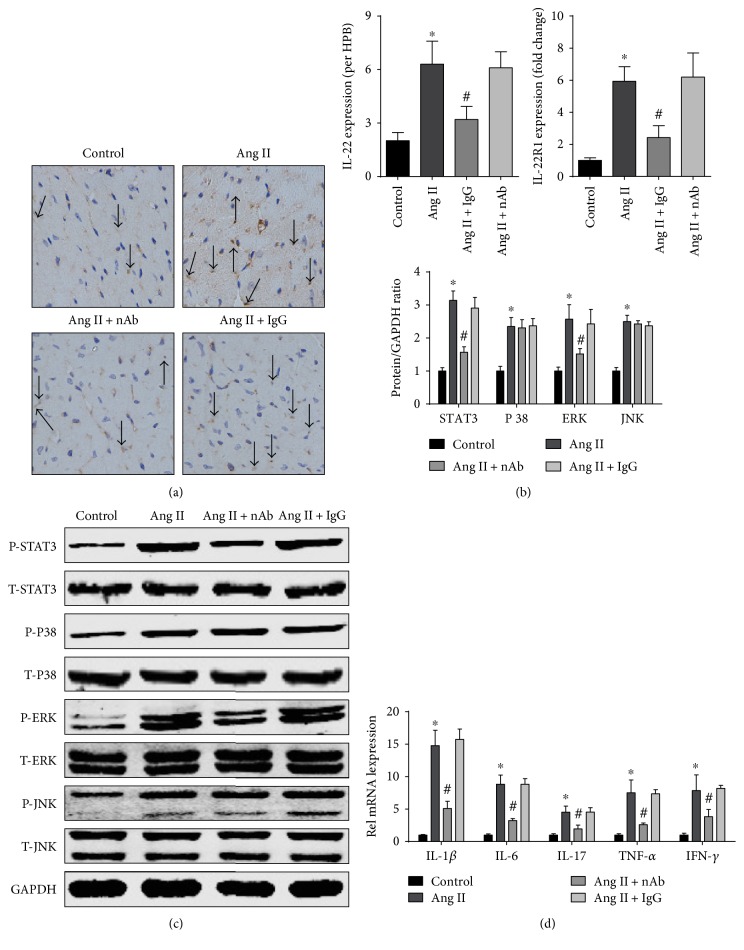
Effect of anti-IL-22 nAb on IL-22/IL-22R1 levels, activation of the STAT3, P38, ERK, and JNK pathways and prohypertrophic inflammatory cytokine mRNA expression in the LV. Effect of IL-22 nAb on (a) IL-22 and (b) IL-22R1 expression, *n* = 3-4 for each group. (c) The effect of the anti-IL-22 nAb on the representative and quantitative expression of phosphorylated and total STAT3, ERK, JNK, and P38 in the LV tissue of each group, *n* = 3-4 for each group. (d) The mRNA expression levels of IL-1*β*, IL-6, IL-17, TNF-*α*, and IFN-*γ* of each group were determined by RT-qPC, *n* = 3-4 for each group. ^∗^*p* < 0.05 versus control. ^#^*p* < 0.05 versus Ang II group.

To investigate the molecular mechanisms of protection against angiotensin II-induced cardiac hypertrophy by anti-IL-22 nAb, the expression of IL-22/IL-22R1, the activation of the STAT3, ERK, JNK, and P38 pathways, and the mRNA levels of prohypertrophic inflammatory cytokines in LV were evaluated. The results showed that angiotensin II infusion increased IL-22 infiltration and IL-22R1 expression in LV, while IL-22 nAb could reduce IL-22 infiltration and IL-22R1 expression (Figures 4(a) and 4(b)). In addition, angiotensin II-induced increase of p-STAT3 and p-ERK was mitigated by anti-IL-22 nAb, and isotype IgG had no effect on this increase. However, the levels of p-P38 and p-JNK were unchanged (Figure 4(c)). The RT-qPCR results showed that anti-IL-22 nAb reduced the angiotensin II-induced release of IL-17, IL-6, IL-1*β*, IFN-*γ*, and TNF-*α* (Figure 4(d)).

### 3.6. IL-22 nAb Protects against an Angiotensin II-Induced Kidney Dysfunction

**Figure 5:**
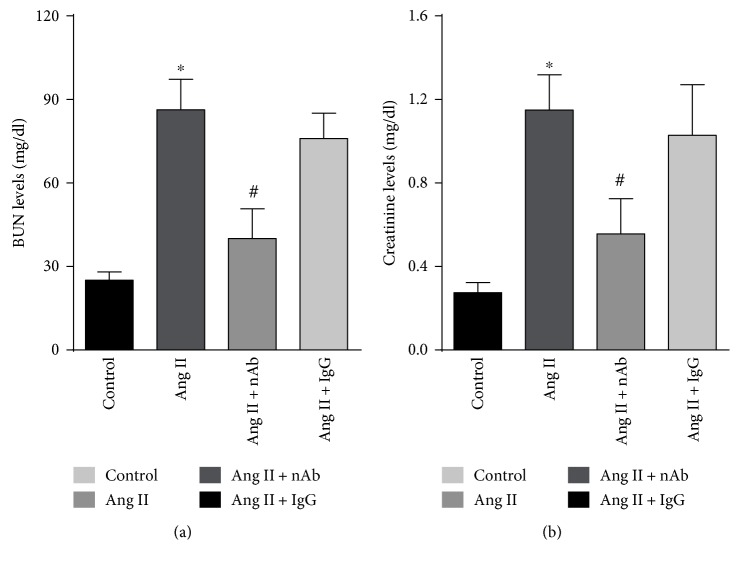
Effect of IL-22 nAb on angiotensin II-induced kidney dysfunction. The (a) serum BUN levels and creatinine levels in control, Ang II, Ang II + nAb, and Ang II + IgG group. *n* = 7-8 for each group. ^∗^*p* < 0.05 versus control. ^#^*p* < 0.05 versus Ang II group.

To determine the effect of IL-22 nAb on angiotensin II-induced kidney dysfunction, the serum blood urea nitrogen and creatinine levels were measured and the results showed that IL-22 nAb significantly reduced serum BUN and creatinine levels in angiotensin II-treated mice, while IgG had no effect on serum BUN and creatinine levels (Figures 5(a) and 5(b)).

### 3.7. IL-22 nAb Alleviates Angiotensin II-Induced Hypertrophy In Vitro

**Figure 6:**
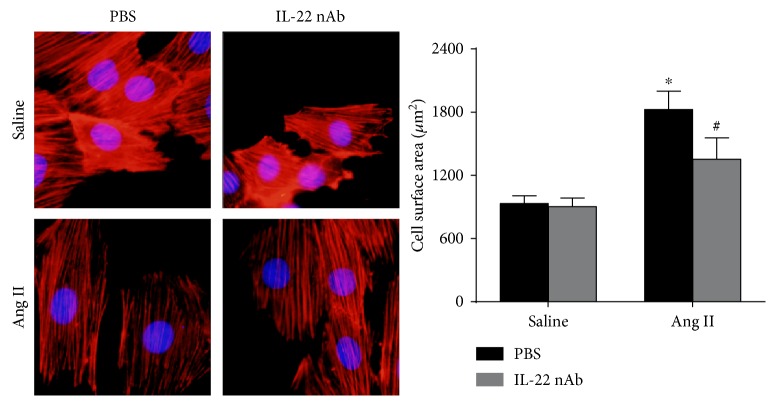
Effect of IL-22 nAb on cardiac hypertrophy in vitro. H9C2 cells were treated with saline + PBS, saline + IL-22 nAb, angiotensin II + PBS, and angiotensin II + IL-22 nAb. *n* > 50 cells for each group. ^∗^*p* < 0.05 versus control. ^#^*p* < 0.05 versus Ang II group.

In order to rule out the effect of blood pressure, whether IL-22 nAb affects angiotensin II-induced hypertrophy in H9C2 cells were detected. The cell surface area of more than 50 H9C2 cells was assessed, and the results showed that IL-22 nAb alleviated angiotensin II-induced hypertrophy in H9C2 cells (Figure 6).

## 4. Discussion

The first aim of the present study was to examine IL-22/IL-22R1 expression in myocardial hypertrophy. Mice received an angiotensin II infusion, and we investigated IL-22 and IL-22R1 levels in heart tissue at different time points. We found that longer angiotensin II infusion resulted in higher levels of hypertrophy marker proteins, which represents a more serious degree of cardiac hypertrophy, with a parallel trend between the progressive increase in IL-22/IL-22R1 protein levels and the seriousness of cardiac hypertrophy. Therefore, the increased IL-22/IL-22R1 protein levels in angiotensin II-induced hypertrophic hearts suggested that IL-22 may participate in myocardial hypertrophy.

IL-22 may have different or even opposite biological functions in different animal disease models. IL-22 accelerates regeneration, and aggravated damage caused by IL-22 was reported in the kidney. Wang et al. found that IL-22 ameliorated renal injury and fibrosis in diabetic nephropathy via downregulating renal NLRP3/caspase-1/IL-1*β* pathway in a mouse model [18], while Weber et al. reported that inhibition of the biological activity of IL-22 by IL-22-binding protein improved host defense and alleviated acute polymicrobial peritonitis-mediated kidney failure [19]. To determine the role of IL-22 in myocardial hypertrophy, mouse anti-IL-22 nAb was used to block the effects of IL-22 on angiotensin II-induced hypertrophy in the present study. The results showed that blockade of IL-22 led to a reduction in the HW/BW ratio, HW/TL ratio, CSA, and protein expression of hypertrophy markers. Additionally, fewer structural abnormalities and reduced systolic and diastolic dysfunction of the LV were observed. These findings suggest a protective effect of anti-IL-22 nAb on angiotensin II-induced hypertrophy and cardiac dysfunction.

The regulation of IL-22 in fibrosis remodeling also has two aspects: IL-22 can have either an antifibrosis or a profibrosis effect in different fibrotic models. Simonian and Liang et al. reported that IL-22 protected against *Bacillus subtilis-* and bleomycin-induced lung fibrosis in mice, respectively [9, 20]. In liver fibrosis, although Wu et al. found that IL-22 activated hepatic stellate cells and mediated fibrogenesis in patients with hepatitis C [21]; most studies reported a protective role of IL-22 in liver fibrosis [22, 23]. In the cardiovascular system, Rattik et al. found that knockout of IL-22 reduced the collagen area of aortic roots in atherosclerosis in mice [13]. Fibrosis, characterized by an increase of collagen and an abnormal distribution, is another integral feature of cardiac hypertrophy. Therefore, we measured the level of cardiac fibrosis in each group. We found that both the fibrotic area and the fibrotic genes, including fibronectin, CTGF, collagen I*α*, collagen III*α*, and TGF-*β*, were significantly increased with angiotensin II infusion, and this profibrosis effect was alleviated by mouse anti-IL-22 nAb. In addition, increases in collagen type I and III, the primary collagen types in the heart, play a crucial role in the progression of myocardial hypertrophy to CHF [24]. The effect of anti-IL-22 on attenuating extracellular fibrosis remodeling is compelling evidence that anti-IL-22 nAb relieves angiotensin II-induced cardiac hypertrophy. While in another study, Guo et al. reported that IL-22 produced reduced myocardial fibrosis in CVB3-induced chronic myocarditis and dilated cardiomyopathy model [25]. Our results seemed to be contradictory with their conclusion. A possible explanation is that the angiotensin II and CVB3 mediated different cardiac fibrosis models.

STAT3 and MAPK are hypertrophy-related signaling pathways. Several studies have indicated that excessive activation of the ERK, JNK, and P38 pathways promotes the progression of angiotensin II-induced cardiac hypertrophy [26–28]. The vast majority of studies have indicated that the excessive activation of the STAT3 pathway promotes cardiac hypertrophy. Interestingly, Gonzalez et al. reported that mice lacking STAT3 Serine 727 phosphorylation showed decreased angiotensin II-induced cardiac hypertrophy, dysfunction, and fibrosis [29]. Gonzales' conclusion was contradictory with others; it may be related to the types of ligands, and may stimulate the intensity, duration, and frequency [30]. The phosphorylation of STAT3 is the primary mediator of IL-22 signaling upon ligation of IL-22-IL-10R2-IL-22R1; in addition, the activation of P38, ERK, and JNK is involved in IL-22 binding. STAT3, ERK, JNK, and P38 pathways could also be activated by angiotensin II. Therefore, to elucidate the molecular mechanisms, the activation of potential signaling pathways, including the ERK, JNK, P38, and STAT3 pathways, was evaluated. Our results showed that the angiotensin II-induced activation of the ERK and STAT3 pathways was mitigated by mouse anti-IL-22 nAb, and the phosphorylation of P38 and JNK was not significantly changed. These results suggested that the anti-IL-22 nAb protected against angiotensin II-induced hypertrophy by downregulating the phosphorylation of the STAT3 and ERK pathways.

Previous studies have demonstrated that the proinflammatory response promoted the development of hypertrophy. Gonzalez et al. reported that angiotensin II-high salt-induced hypertrophy was prevented in IL-6 deletion mice [29]. Marko et al. reported that knockout of the IFN-*γ* receptor reduced cardiac hypertrophy [30], and a similar effect was observed after IL-17R blockade and in mice lacking TNF-*α* [31, 32]. Although the effect of IL-1*β* on angiotensin II-dependent cardiac hypertrophy is unknown, data from clinical settings and experiments have indicated that IL-22-IL-22R1-IL-10R2 binding can regulate IL-6, IL-17, IL-1*β*, TNF-*α*, and IFN-*γ* expression [33]. To determine whether IL-22 participates in angiotensin II-induced hypertrophy by regulating inflammatory cytokine expression, we measured the mRNA expression of these prohypertrophic inflammatory cytokines in the LV. Our data showed that anti-IL-22 nAb treatment resulted in lower mRNA expression of IL-17, IL-6, IL-1*β*, TNF-*α*, and IFN-*γ* in the LV. These results suggested that mouse anti-IL-22 participates in angiotensin II-induced hypertrophy by reducing levels of inflammatory mediators.

Previous studies demonstrated that as the subset of CD4^+^ helper (Th) cells, Th1 and Th17 and their cytokine secretion profile IFN-*γ* and IL-17 were critical in the angiotensin II-induced hypertension model [34, 35]. IL-22 is mainly derived from Th22 cell which is the new subset of CD4^+^ cells, whether Th22/IL-22 affects angiotensin II-induced hypertension is still unknown. We could not rule out whether the antihypertrophy effect of IL-22 nAb was due to antihypertensive effect in angiotensin II-treated mice; therefore, we detected the effect of IL-22 nAb in angiotensin II-induced hypertrophy in vitro and we found that treatment with IL-22nAb protected against angiotensin II-induced hypertrophy in H9C2 cells. These results suggested that IL-22 nAb could indeed play a protective role in angiotensin II-induced hypertrophy, while whether IL-22 affect angiotensin II-induced hypertension remained unknown and further studies were needed.

The results of the present study demonstrated that anti-IL-22 nAb exerts a protective effect against the progression of cardiac hypertrophy induced by angiotensin II by upregulating the expression of inflammatory mediators, but this study has several limitations. First, the anti-IL-22 nAb reduced the activation of the STAT3 and ERK pathways induced by angiotensin II. However, we did not use a STAT3 or ERK inhibitor to examine the effect on cardiac hypertrophy and the expression of downstream prohypertrophic inflammatory cytokines. In addition, the mRNA levels of several inflammatory cytokines which could promote cardiac hypertrophy were tested. We did not neutralize some of these cytokines and observed the changes of other cytokines in the hypertrophy model; therefore, whether these inflammatory cytokines are directly or indirectly regulated by IL-22 is unknown. In conclusion, IL-22 may be a novel pharmacotherapeutic strategy for the treatment of cardiac hypertrophy induced by angiotensin II to limit the progression of heart failure, downregulate the circulatory IL-22 level, prevent the effects of IL-22 and IL-22R on the heart, and reduce the excessive activation of the STAT3 or ERK pathway.

## Figures and Tables

**Table 1:** RT-PCR primers used.

Gene	Forward primer (5′-3′)	Reverse primer (5′-3′)
FN CTGF Col I*α* Col III*α* TGF-*β* IL-6 IL-1*β* TNF-*α* IFN-*γ*	CCGGTGGCTGTCAGTCAGA TGTGTGATGAGCCCAAGGAC TGGTACATCAGCCCGAAC ACGTAGATGAATTGGGATGCAG TGCTTCAGCTCCACAGAGAA AGTTGCCTTCTTGGGACTGA GGGCCTCAAAGGAAAGAATC CCCAGGGACCTCTCTCTAATC ACTGGCAAAAGGATGGTGAC	CCGTTCCCACTGCTGATTTATC AGTTGGCTCGCATCATAGTTG GTCAGCTGGATAGCGACA GGGTTGGGGCAGTCTAGTC TGGTTGTAGAGGGCAAGGAC TCCACGATTTCCCAGAGAAC TACCAGTTGGGGAACTCTGC ATGGGCTACAGGCTTGTCACT TGAGCTCATTGAATGCTTGG
IL-17 IL-22R1	TCCAGAAGGCCCTCAGACTA CTACGTGTGCCGAGTGAAGA	AGCATCTTCTCGACCCTGAA AAGCGTAGGGGTTGAAAGGT
GAPDH	AACTTTGGCATTGTGGAAGG	CACATTGGGGGTAGGAACAC

FN: fibronectin; Col: collagen.

**Table 2:** Echocardiographic data of each group.

Groups	Control	Ang II	Ang II + nAb	Ang II + IgG
HR (bpm)	533.4 ± 58.5	529.5 ± 39.8	511.9 ± 50.2	526.9 ± 34.5
LVEDD (mm)	3.51 ± 0.23	5.14 ± 0.24^∗^	4.31 ± 0.34^**#**^	5.09 ± 0.30
LVESD (mm)	2.67 ± 0.28	3.86 ± 0.19^∗^	3.03 ± 0.20^**#**^	3.71 ± 0.35
LVPWD (mm)	0.71 ± 0.04	0.78 ± 0.04^∗^	0.72 ± 0.03^**#**^	0.79 ± 0.03
IVSD (mm)	0.65 ± 0.03	0.79 ± 0.03^∗^	0.72 ± 0.03^**#**^	0.79 ± 0.03
EF (%)	74.57 ± 1.99	49.75 ± 2.82^∗^	63.38 ± 4.89^**#**^	48.38 ± 5.12
FS (%)	44.29 ± 2.31	25.38 ± 2.34^∗^	35.38 ± 3.31^**#**^	25.63 ± 2.29

*n* = 8 for each group. ^∗^*p* < 0.05 versus control. ^#^*p* < 0.05 versus Ang II group.
