# Erratum to “Anti-Interleukin-22-Neutralizing Antibody Attenuates Angiotensin II-Induced Cardiac Hypertrophy in Mice”

**DOI:** 10.1155/2018/2586494

**Published:** 2018-08-26

**Authors:** Jing Ye, Ling Liu, Qingwei Ji, Ying Huang, Ying Shi, Lei Shi, Jianfang Liu, Menglong Wang, Yao Xu, Huimin Jiang, Zhen Wang, Yingzhong Lin, Jun Wan

**Affiliations:** ^1^Department of Cardiology, The People's Hospital of Guangxi Zhuang Autonomous Region, Nanning, China; ^2^Department of Cardiology, Hubei Key Laboratory of Cardiology, Renmin Hospital of Wuhan University, Cardiovascular Research Institute, Wuhan University, Wuhan 430060, China; ^3^Emergency & Critical Care Center, Beijing Institute of Heart, Lung, and Blood Vessel Diseases and Beijing Anzhen Hospital, Capital Medical University, Beijing 100029, China

In the article “Anti-Interleukin-22-Neutralizing Antibody Attenuates Angiotensin II-Induced Cardiac Hypertrophy in Mice” [1], there was an error in Figure 1(a), where the IL-22, *β*-MHC, and GAPDH bands were triplicated, due to a production error. The corrected figure is shown below.

## Figures and Tables

**Figure 1:**
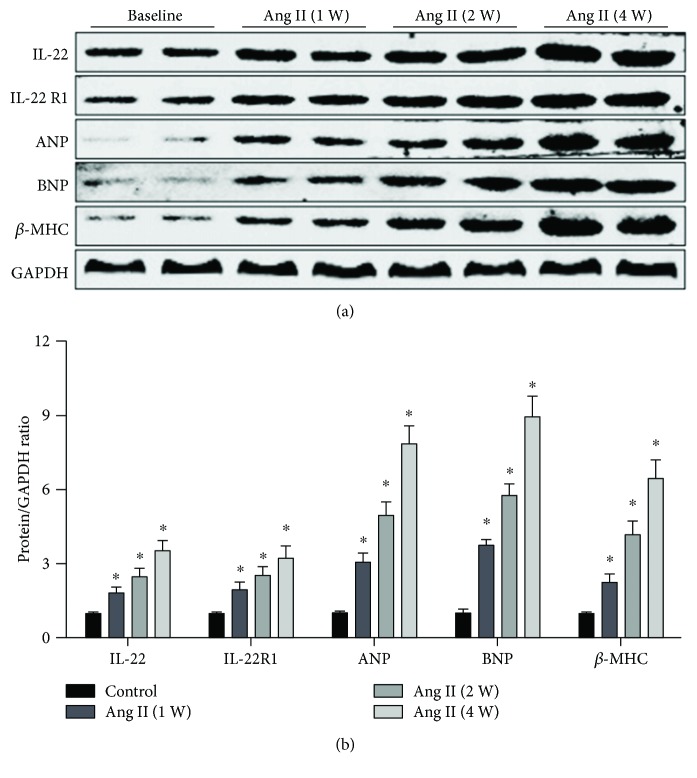
Effect of chronic angiotensin II infusion on IL-22/IL-22R1 protein levels. (a) Representative and (b) quantitative expression of IL-22, IL-22R1, ANP, BNP, and *β*-MHC in LV tissue of baseline and angiotensin II-infused mice. *n* = 4 for each group. ^∗^*p* < 0.05 versus baseline level.
